# Predicting Aromatic Amine Mutagenicity with Confidence: A Case Study Using Conformal Prediction

**DOI:** 10.3390/biom8030085

**Published:** 2018-08-29

**Authors:** Ulf Norinder, Glenn Myatt, Ernst Ahlberg

**Affiliations:** 1 Swetox, Karolinska Institutet, Unit of Toxicology Sciences, SE-151 36 Södertälje, Sweden; 2 Dept. Computer and Systems Sciences, Stockholm Univ., Box 7003, SE-164 07 Kista, Sweden; 3 Leadscope, 1393 Dublin Road, Columbus, OH 43215, USA; gmyatt@leadscope.com; 4 Drug Safety and Metabolism, Innovative Medicines and Early Development Biotech Unit, AstraZeneca R&D Gothenburg, SE-431 83 Mölndal, Sweden; Ernst.AhlbergHelgee@astrazeneca.com

**Keywords:** aromatic amines, mutagenicity, conformal prediction, confidence, random forest

## Abstract

The occurrence of mutagenicity in primary aromatic amines has been investigated using conformal prediction. The results of the investigation show that it is possible to develop mathematically proven *valid* models using conformal prediction and that the existence of uncertain classes of prediction, such as *both* (both classes assigned to a compound) and *empty* (no class assigned to a compound), provides the user with additional information on how to use, further develop, and possibly improve future models. The study also indicates that the use of different sets of fingerprints results in models, for which the ability to discriminate varies with respect to the set level of acceptable errors.

## 1. Introduction

Primary aromatic amines (PAAs) are present in a wide variety of chemicals including drugs, cosmetics, dyes, plastics, food products, tobacco and pesticides. They are commonly used in the manufacture of pharmaceuticals; however, PAAs are a structural alert for mutagenicity. This PAA alert has been extensively reviewed in the literature and there is a clear association between this class of compounds and mutagenic activity [1,2,3,4,5,6,7,8]. In drug discovery, mutagenicity is an issue that needs to be avoided. Detection of mutagenicity during early stages of drug discovery is of considerable importance in order avoid the development of potentially harmful drugs. Computational methods for detecting mutagenicity are mainly of two types: approaches based on structural alerts [9,10,11] or statistically based quantitative structure–activity relationships (QSARs) [11,12].

Several models and software are currently available for the prediction of mutagenicity such as CAESAR [13], Derek Nexus [14], Lazar [15], Leadscope [11,16], MultiCASE [17] and OECD Toolbox [18]. Quantitative structure–activity relationship (QSAR) models are empirical models in that an empirical relationship between descriptors and experimental response is sought. As a consequence these models have a certain associated applicability domain where reliable predictions can be expected. This has been recognized by OECD in their guidance document for QSAR model development [19].

In this study, the conformal prediction method is applied for predicting mutagenicity of PAAs using Leadscope fingerprints [16]. Conformal prediction is a framework for deriving machine learning models, e.g., QSAR models, resulting in predictions with a predefined significance level, i.e., error rate [20]. A conformal predictor will make *valid* predictions on new compounds corresponding to the user-defined significance level (the percentage of acceptable predictions errors). This includes taking applicability domain into account as part of the framework [21]. The significance level can be altered, which allows for flexibility and adaption to the risks that the user is willing to accept.

## 2. Results

The purpose of the investigation is to study aromatic amine mutagenicity using a new QSAR method called conformal prediction [20,21]. This method allows the user to set a predefined error level (percentage of errors for each experimental class) termed significance level to which the developed in silico model will comply under standard QSAR requirements such as exchangeability. That is the new test data behaves similarly to the training data. In such cases, the derived conformal prediction model is *valid*. *Validity* in this investigation refers to conformal prediction nomenclature and is described in the section “Model generation” and in Equation (1). For a 2-class classification problem, like the one studied in this investigation, the outcome from conformal prediction is not only classifying new test compounds to one of the two original experimental outcomes (mutagenic and nonmutagenic) but also to classify them as belonging to class *both* or class *empty*. Classifying a new test compound as *both* indicates that the prediction is within the applicability domain of the model but that the model cannot distinguish between the two experimental outcomes. Classifying a new test compound as *empty* means that the new compound is outside the applicability domain of the model and no classes can be assigned. *Efficiency*, another conformal prediction defined term, means the number of compounds predicted as either mutagenic or nonmutagenic, that is, not classified as *both* or *empty*, in relation to the total number of compounds predicted by the model.

### 2.1. Conformal Prediction Model Based on Leadscope Fingerprints

**Figure 1:**
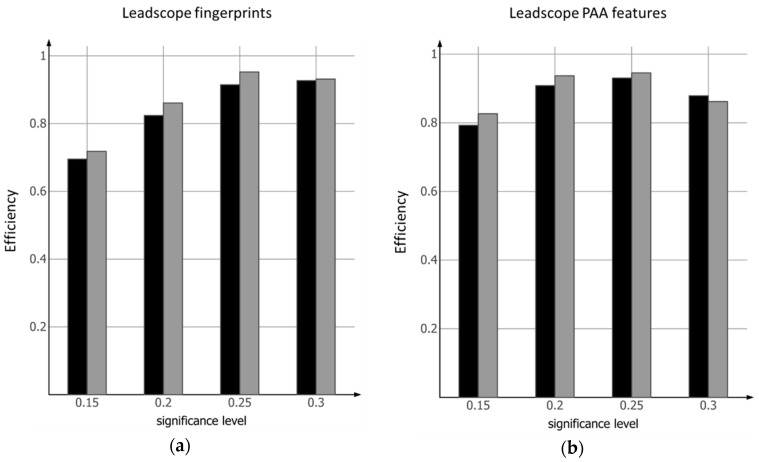
*Efficiency* (single class predictions) for the models: (**a**) based on Leadscope fingerprints; (**b**) based on Leadscope PAA features. Black bars, internal test sets; grey bars, external test sets.

Within small statistical fluctuations, all models are *valid,* for both the mutagenic as well as the nonmutagenic classes at the different significance levels (Table 1). The *efficiency* increases with increasing significance levels as the number of compounds classified as *both* decreases and reaches a maximum for the external test set at the 0.25 level. After that, the *efficiency* starts to decrease as the number of compounds classified as *empty* starts to appear (Figure 1a). The predictive performance for both classes with respect to *validity* is similar for the internal and the external test set. Also, more traditional performance measures, such as balanced accuracy, Cohen’s Kappa and Matthews correlation coefficient show the same balance between the two test sets.

### 2.2. Conformal Prediction Model Based on Leadscope PAA Features

Similar to the models based on Leadscope fingerprints, the corresponding model based on more specifically derived PAA features (defined in Section 4.2.2) are all *valid* for both the mutagenic as well as the nonmutagenic classes at the different significance levels (Table 1). Also for these models the *efficiency* increases with increasing significance level as the number of compounds classified as *both* decreases and reaches a maximum at the 0.25 level, after which the number of compounds classified as *empty* starts to appear (Figure 1b). The *efficiency* decreases slightly more for the models at the 0.30 significance level compared to the models based on Leadscope fingerprints. Again, the predictive performance for both the mutagenic as well as the nonmutagenic class with respect to *validity*, balanced accuracy, Cohen’s Kappa and Matthews correlation coefficient is similar and balanced with respect to internal and external test sets.

## 3. Discussion

One of the advantages of conformal prediction is the mathematically proven *validity* [20] of the models given exchangeability of the dataset. The latter constitutes the basis for any successful QSAR model but using conformal prediction the *validity* is guaranteed. Since all models derived in this work are *valid* for both (mutagenic and nonmutagenic) classes it is safe to state that the data is exchangeable. However, the *efficiency* and, as a direct consequence, the applicability domain of the two sets of models based on Leadscope fingerprints and Leadscope PAA features are slightly different. This is related to the fact that compounds are predicted as *both* or *empty* depending on the set significance level. Compounds predicted as *both*, i.e., predicted as both mutagenic as well as nonmutagenic, are defined by conformal prediction to be correctly predicted. These compounds are also similar enough to the training (calibration) set compounds for the two classes to have reliable predictions and, thus, considered to be within the applicability domain of the model. For compounds assigned to the *empty* class the situation is the opposite, i.e., these compounds are dissimilar to the training (calibration) set compounds, and considered outside the applicability domain of the model. Figure 1a,b show that Leadscope PAA features are more effective for distinguishing mutagenic and nonmutagenic PAAs at low significance levels (0.15 and 0.20), i.e., high confidence levels (0.85 and 0.80) compared to the more general Leadscope fingerprints. The two sets of fingerprints have approximately equal *efficiency* at the 0.25 significance level. Leadscope fingerprints are more efficient at the 0.30 level compared to Leadscope PAA features where, for both sets of fingerprints, all non-single class classified compounds belong to the *empty* class. However, at the 0.20 significance level, where both sets of fingerprints are equally efficient, the reasons for this equality are completely opposite. The model based on Leadscope fingerprints classify almost all non-single class predicted compounds as *both* (one exception) while the model based on Leadscope PAA features classify all non-single class predicted compounds as *empty*. These results show that Leadscope PAA features are more effective at distinguishing mutagenic and nonmutagenic compounds at low acceptable error rates, especially at the lowest error rate of 15%; however, compared with the more general Leadscope fingerprints there is a more restricted domain of applicability when allowing for higher levels of acceptable errors. The results are in line with the reason for developing the Leadscope PAA features namely to create new, more compound class focused fingerprints, with increased ability for distinguishing between mutagenic and nonmutagenic compounds such as the PAA class of compounds.

An additional favorable property when using conformal prediction is the graceful handling of the minority class [22,23,24], in this investigation the nonmutagenic class. Although the class imbalance for the aromatic amine dataset is fairly small (mutagenic/nonmutagenic ratio ≈ 2:1) the sensitivity and specificity values are quite balanced for the single label predicted compounds. This, in turn, means that the number of false positives (FP) are kept low and at a level corresponding to the same ratio with respect to false negatives (FN) as the dataset in general, namely, around 2:1 (FN:FP). An illustrative example of this is the Leadscope fingerprints and Leadscope PAA features based models with both validity and high efficiency at the 0.2 significance level. For the former set of models the average values across the 50 external test sets for true positives (TP), FN, true negatives (TN) and FP are 125, 39, 60 and 17, respectively, and the corresponding values for the latter models are 141, 35, 69 and 18 respectively. At this significance level the FN:FP ratio for the Leadscope fingerprints and Leadscope PAA features based models is 2.29 and 1.94, respectively, indicating that no serious increase with respect to FP has occurred. The reason for this exception, in comparison to some other approaches, is the class dependent recalibration of the original random forest (RF) class probabilities using the respective mutagenic and nonmutagenic calibration set and the subsequent conformal prediction *p*-value calculation forming the basis for the final class assignment (prediction) into one of the four possible choices (mutagenic, nonmutagenic, *both*, *empty*).

In contrast to most classical binary classification schemes, the existence of the two additional classes *both* and *empty,* also provides the user of these models with additional information on how to act. If a desirable, or even acceptable, level of error is at the 0.15 significance level the efficiencies, on average, are approximately 0.68 and 0.79 for the models based on Leadscope fingerprints and Leadscope PAA features, respectively. If these efficiencies are not sufficient for the decision to be taken then additional new input information (characterization of the compounds), e.g., of more biochemical nature, is needed in order to increase the efficiencies. On the other hand, if the *efficiency* is too low mainly because of too many *empty* predictions, as is the case for the models at the 0.3 significance level, then the models should be upgraded with these compounds once the experimental classes have been experimentally determined. Thus, if the *efficiency* is not high enough at the desired significance level then new information charactering the investigated compounds must be added. This new information may be of more biological nature, e.g., assay information of some sort, which permits resolution of the *both* class so that the *efficiency* is increased to an acceptable level for the decision, while maintaining the error rate at the desired significance level.

## 4. Materials and Methods

### 4.1. Primary Aromatic Amine Dataset

A database of PAAs was generated from: (1) the training sets used to build (Q)SAR models at the US Food and Drug Administration’s (FDA) Center for Drug Evaluation and Research with Research Collaboration Agreement (RCA) partners (RCA-QSAR) [25,26] and (2) the Leadscope 2015 SAR database [27]. The RCA-QSAR bacterial mutagenicity database includes chemicals containing a PAA, with calls derived from multiple *Salmonella* and *Escherichia coli* strains [28]. This data is nonproprietary and includes information harvested from FDA approval packages and the published literature. The Leadscope 2015 SAR Genetox Database contains chemicals with graded bacterial mutagenesis calls from a variety of sources including: (1) the FDA Center for Food Safety and Applied Nutrition (CFSAN) Food Additive Resource Management system (FARM) and Priority based Assessment of Food Additives (PAFA) database [29]; (2) the US FDA’s Center for Drug Evaluation and Research (CDER) pharmacology/toxicology reviews for new drug application (NDA), available as approval packages [30]; (3) the Chemical Carcinogenicity Research Information System (CCRIS) [31]; (4) the National Toxicology Program’s (NTP) genetic toxicology database [32]; (5) the Tokyo-Eiken database [33]; (6) other publications; and (7) chemicals donated from pharmaceutical companies. In total 936 Primary aromatic amines (PAA) were used containing 630 active (mutagenic) and 306 inactive (nonmutagenic) compounds with an active to inactive ratio of approximately 2:1 (2.06:1).

### 4.2. Leadscope Molecular Descriptors

#### 4.2.1. Leadscope Fingerprints

The general Leadscope fingerprints were used as a molecular descriptor set. The total set includes over 27,000 unique predefined substructures organized into 14 high level classes: amino acids, heterocycles, bases, nucleosides, naphthalenes, benzenes, natural products, carbocycles, peptidomimetics, carbohydrates, pharmacophores, elements, protective groups, functional groups, and spacer groups [16].

#### 4.2.2. Leadscope PAA Features

A process, referred to as SAR fingerprinting, was used to derive the set of PAA features [34]. The first step in the process was to systematically define a set of 591 PAA substructure searches or features that cover different aniline substitution patterns as well as different fused and heteroaromatic PAAs. These features were then matched against a series of public and proprietary databases containing experimentally tested chemicals with bacterial mutagenicity data. For each database, this matching process involved performing a series of substructure searches to match each of the features using the Leadscope software. The number of experimentally determined mutagenic (active) and nonmutagenic (inactive) compounds were calculated for each feature. These counts were then summed across all the public and proprietary databases. The total number of mutagenic and nonmutagenic chemicals matching each feature was calculated. This information was then used to determine a subset of these features that activate or deactivate PAA mutagenicity, as outlined in [34]. This subset of 157 features makes up the fixed set of PAA features used in this exercise (i.e., the PAA fingerprint).

### 4.3. Conformal Prediction

#### 4.3.1. Model Generation

**Figure 2:**
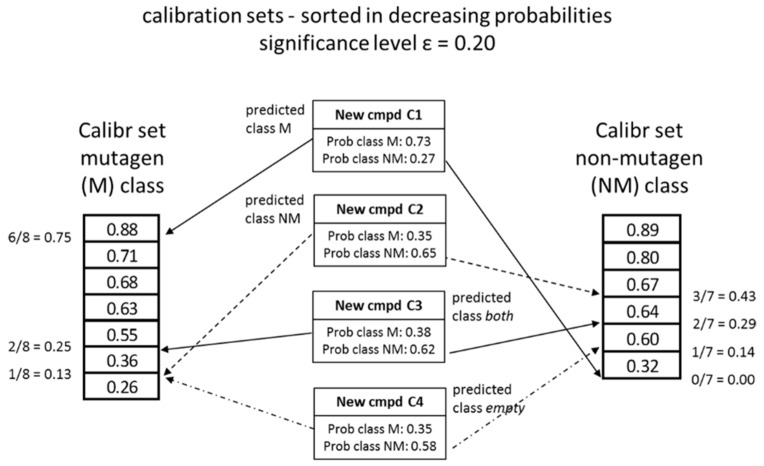
Calculation of conformal prediction *p*-values and class assignments. Abbreviations: cmpd, compound; Prob, probability; Calibr, calibration.

A conformal predictor will make *valid* predictions according to a user defined significance level. The significance level is the percentage of, to the user, acceptable errors that the model may commit. In a binary classification problem, a set of class labels are assigned to new compounds by comparing them to calibration sets classifications with known labels (mutagenic and nonmutagenic). These calibration sets are randomly selected from the training set (see following section of model validation). If the prediction outcome for a new compound is higher than the set significance level, i.e., similar enough to the corresponding predictions for the calibration set compounds for classes M (mutagenic) and NM (nonmutagenic), respectively, the new compound is assigned that class label. This procedure is performed for each new compound and each label (class) in the dataset. Consequently, for a binary classification problem there are four possible outcomes. A new instance can be labelled with either of the two classes, assigned both labels (*both* classification) or none of the labels (*empty* classification). The procedure is illustrated in Figure 2 and described below in more detail.

The percentage of trees in the random forest ensemble predicting each of the two classes (class probability) is used as a conformal prediction similarity (conformity) measure. Conformal prediction assigns classes to new compounds by comparing the class probability against the corresponding sorted list of class probabilities for the calibration set associated with each RF model.

The predicted class probabilities for classes M and NM of the new compound is placed in the sorted list of calibration set probabilities for classes M and NM, respectively, adding one compound to the list for each class. The position of the new compound in each of these two sorted lists is determined and the fraction with lower probabilities is calculated. This fraction is compared to the corresponding significance level set by user. For a new compound to be assigned a class the calculated fraction must be larger or equal to the set significance level.

The four possible outcomes from a binary classification task is illustrated in Figure 2 and described in the following section:

New compound C1 has predicted class probabilities for class M and NM of 0.73 and 0.27, respectively. Placing these probabilities in the corresponding sorted calibration set list of probabilities results in positions 6 for class M and position 1 for class NM. The corresponding calculated fractions, called conformal predictions *p*-values, are 0.75 and 0.0, respectively. The set significance level in this example is 0.20 and new compound C1 can be assigned to class M (0.75 ≥ 0.20) but not to class NM (0.0 < 0.20). Similarly new compound C2 can only be assigned to class NM. For the two remaining new compounds C3 and C4 the situation is somewhat different. For new compound C3, the calculated fractions for both classes are above the set significance level and, consequently, this compound is assigned to both class M and NM (the *both* class). For new compound C4 the situation is the opposite and both calculated fractions are below the set significance level and new compound C4 cannot be assigned to any of the two classes by the model (the *empty* class). For new compound C4 it should be noted, for clarity, that 7 decision trees did not give a class assignment, e.g., the resulting leaf node was unable to provide a majority class vote.

For more examples on how conformal prediction is carried out, we refer the reader to [21].

The performance of a conformal predictor is often measured by its *validity* and *efficiency*. A conformal predictor is *valid* if the percentage of errors does not exceed the set significance level. In conformal prediction a prediction is considered correct if it includes the correct predicted class label, which means that *both* predictions are always correct and, vice versa, *empty* predictions are never correct (i.e., always erroneous). Thus, the *validity* for class A (M or NM) is the sum of the number of correct class predictions and the number of *both* class predictions for class A compounds divided by total number of predicted class A compounds:

*Validity* (A) = (#correct_predictions[A] + #*both*_predictions[A])/#total_predicted_compounds[A] (1)

The *efficiency* in conformal prediction is calculated as the percentage of the total number of single class predictions, regardless of whether they are correct or not, in relation to the total number of predicted compounds. Thus, if 75% of the predicted compounds are assigned to either class M or NM, respectively, then the efficiency of the conformal prediction model is 0.75. The rest of the compounds (25%) are, consequently, predicted as *both* or *empty* class compounds.

Many times a trade off in conformal prediction is that between the *validity* of the model and the *efficiency*.

We have used the RF algorithm [35] for deriving the underlying models in our conformal predictors. The models were developed using Python, Scikit-learn [36] version 0.17, and the nonconformist package [37] version 1.2.5. Binary classification models were built based on RF using the Scikit-learn RandomForestClassifier with 100 trees and all other options set at the default value.

#### 4.3.2. Model Validation

**Figure 3:**
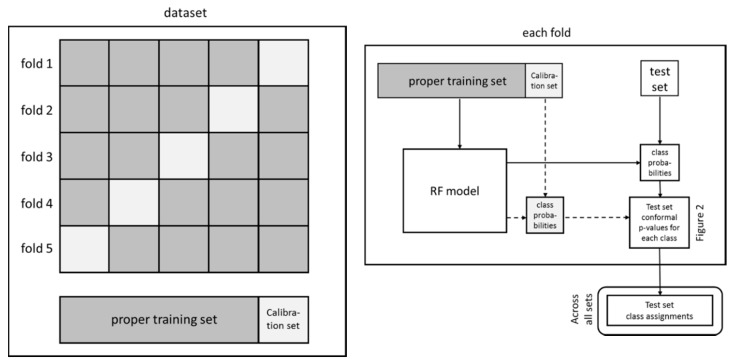
The fivefold cross-validation procedure using conformal prediction (cross-conformal prediction).

The data set was randomly divided into a training (70%, 656 compounds) and an external test (30%, 280 compounds) set. This procedure was repeated 50 times to generate 50 pairs of random training and external test sets. The cross-conformal prediction method described by Sun et al. [38] was then applied and each training set was further divided into a proper training set and calibration set, 80% and 20%, respectively, using five random cross-validation folds (Figure 3, left part). The proper training set was used for deriving the RF model and the calibration set for predicting the conformal prediction *p*-values of the test set. For each of the 5 folds the *p*-value predictions on the corresponding external test set were stored and the results over all 50 test sets were used for class assignments in accordance with the set significance levels (Figure 2) and reported in Table 1. Also, from each of the five proper training sets generated by the fivefold cross-validation an internal training (80%) and internal test set (20%) was randomly selected. The latter set was then predicted and assigned classes using the same procedure as for the external test set over all 50 internal test sets.

In order to quantify the quality of the derived models the following classification metrics were used [39]:

Sensitivity (true positive rate) = TP/(TP + FN)(2)

Specificity (true negative rate) = TN/(TN + FP)(3)

Balanced accuracy (BA) = (Sensitivity + Specificity)/2(4)

Kappa = (po − pe)/(1 − pe)(5)

Matthews correlation coefficient (MCC) = (TP × TN − FP × FN)/ √[(TP + FP)(TP + FN)(TN + FP)(TN + FN)](6)

where:

TP = true positives (no. of mutagenic compounds correctly classified as mutagenic);

FP = false positives (no. of nonmutagenic compounds incorrectly classified as mutagenic);

TN = true negatives (no. of nonmutagenic compounds correctly classified as nonmutagenic);

FN = false negatives (no. of mutagenic compounds incorrectly classified as nonmutagenic);

po = observed accuracy = (TP + TN)/(TP + FP + TN + FN);

pe = expected accuracy = [(TP + FN)(TP + FP) + (TN + FP) (TN + FN)]/[(TP + FP + FN + TN)^2^].

## 5. Conclusions

Accurate predictions of PAAs are important for the development of new products, including pharmaceuticals. Prediction methods that both minimize false positive and false negative predictions are critical since false positives may lead to further unnecessary experimental testing (to establish whether the compound is mutagenic) or force a new research direction to be considered whereas, false negatives could compromise safety of those exposed to the chemical if not detected.

This study using conformal prediction highlights several interesting aspects with respect to in silico model building:

- It is possible to develop mathematically proven *valid* models using the conformal prediction framework for predicting mutagenicity of primary aromatic amines.
- The user of the models can set the acceptable level of errors.
- The existence of uncertain classes of prediction (*both* and *empty*) provides the user with information on how to use, further develop or possibly improve future models.
- The use of different sets of fingerprints results in models where the ability to discriminate varies with respect to the set level of acceptable errors.

## Figures and Tables

**Table 1:** Results from the fivefold cross-conformal prediction across 50 test sets.

Descriptors ^a^	Set ^b^	No Of Compounds Per Set	Significance Level ^c^	Validity Mutagenic Class	Validity Nonmutagenic Class	Efficiency ^d^	BA ^e^	Kappa ^f^	MCC ^g^	Sensitivity ^h^	Specificity ^h^	Percentage Class *Both* ^i^	Percentage Class *Empty* ^j^
LS fingerprints	internal	656	0.15	0.851	0.849	0.695	0.537	0.543	0.784	0.786	0.782	30.5	0
LS fingerprints	external	280	0.15	0.857	0.871	0.718	0.581	0.589	0.809	0.804	0.814	28.2	0
LS fingerprints	internal	656	0.20	0.803	0.797	0.824	0.485	0.493	0.759	0.764	0.753	17.5	0.2
LS fingerprints	external	280	0.20	0.794	0.818	0.861	0.508	0.518	0.773	0.763	0.782	13.9	0
LS fingerprints	internal	656	0.25	0.753	0.746	0.914	0.452	0.460	0.742	0.746	0.738	7.1	1.4
LS fingerprints	external	280	0.25	0.748	0.766	0.952	0.467	0.476	0.751	0.742	0.759	4.2	0.6
LS fingerprints	internal	656	0.30	0.707	0.697	0.927	0.449	0.457	0.741	0.742	0.740	1.6	5.7
LS fingerprints	external	280	0.30	0.696	0.717	0.931	0.478	0.490	0.759	0.741	0.776	0.2	6.7
LS PAA features	internal	656	0.15	0.853	0.851	0.793	0.596	0.601	0.813	0.815	0.811	20.7	0
LS PAA features	external	280	0.15	0.857	0.855	0.826	0.625	0.630	0.826	0.826	0.826	17.4	0.0
LS PAA features	internal	656	0.20	0.802	0.795	0.908	0.541	0.548	0.786	0.790	0.782	8.5	0.7
LS PAA features	external	280	0.20	0.810	0.802	0.937	0.567	0.573	0.798	0.802	0.794	5.9	0.4
LS PAA features	internal	656	0.25	0.753	0.746	0.930	0.532	0.539	0.782	0.783	0.781	2.3	4.7
LS PAA features	external	280	0.25	0.758	0.748	0.945	0.559	0.565	0.794	0.798	0.791	0.3	5.2
LS PAA features	internal	656	0.30	0.703	0.697	0.879	0.556	0.563	0.794	0.795	0.794	0.3	11.9
LS PAA features	external	280	0.30	0.706	0.705	0.862	0.608	0.613	0.818	0.820	0.816	0.0	13.8

^a^ Leadscope descriptors (see Section 4.2.1 and Section 4.2.2), ^b^ Internal and external test set (see Section Model validation for a description), ^c^ Significance level (% acceptable errors), ^d^ Percentage of single class (mutagenic or nonmutagenic) predictions, ^e^ Balanced accuracy, ^f^ Cohen’s Kappa, ^g^ Matthews correlation coefficient, ^h^ Sensitivity and specificity are calculated using only single label classified compounds, ^i^ Percentage of compounds predicted as *both*, ^j^ Percentage of compounds predicted as *empty*. Abbreviations: LS, general Leadscope; PAA, primary aromatic amine.
